# Sulfonated Hydrogel Formed via CO_2_-in-Water Emulsion: Potential in Antibiotic Removal

**DOI:** 10.3390/gels9090703

**Published:** 2023-08-31

**Authors:** Kaibo Xu, Liqin Cao

**Affiliations:** 1 Key Laboratory of Oil and Gas Fine Chemicals, College of Chemistry, Ministry of Education & Xinjiang Uygur Autonomous Region, Xinjiang University, Urumqi 830017, China; mrxu125@foxmail.com; 2 School of Science, Xihua University, Chengdu 610039, China

**Keywords:** high-internal-phase emulsion, CO_2_-in-water, chitosan oligosaccharides, monolith, porous, hydrogel

## Abstract

Herein, a green, carbon dioxide-in-water high-internal-phase emulsion (C/W HIPEs) was developed and stabilized with polyvinyl alcohol (PVA) for the formation of chitosan oligosaccharide/poly(acrylamide-co-sodium 4-styrene sulfonate) [COS/P(AM-co-SSS)] monolithic porous hydrogel. The obtained monolith was characterized via FT-IR and SEM. The SEM patterns depicted that the monoliths were interconnected, the void sizes were 78.5 µm, and the interconnected pore throats were 28 μm approximately. Mechanical measurement results indicated that the maximum compress stress of the monolith could reach 334.4 kPa at 90% strain, and it exhibited good mechanical stability. After 200 cycles of compression, it could still recover its original shape without cracking. The obtained COS-based monolith was selected to remove tetracycline (TC) for evaluating the adsorptive features of the interpenetrating pore-containing monolith. The monolithic COS/P(AM-co-SSS) hydrogel behaved with strong antibiotic adsorption capacity (1600.4 mg/g for TC). The adsorption process agreed well with the pseudo-second-order kinetic and Langmuir isothermal models. In addition, the porous monolith had a strong electrostatic force on TC according to the thermodynamic study. This work provides a green route for the development of novel monolithic hydrogels and highlights its potential application in the treatment of antibiotic-containing wastewater.

## 1. Introduction

Porous polymers with many functional groups have attracted attention due to their wide applications as catalyst supports, as adsorbents, as tissue scaffolds, and in controlled drug release [1,2,3]. So far, various methods have been applied to prepare porous macromolecules. Shen et al. synthesized ordered macro-microporous material by sacrificing a polystyrene template [4]. Song et al. briefly increased the porosity (resin/sugar) above 95% on the original basis via a stereolithography-based sugar foaming route [5]. Mu et al. reported and synthesized 3D porous structural material using a 3D printing method [6]. The effectiveness of emulsion templating technologies has been verified to form a highly porous and permeable polymeric material with well-defined porosity [1]. A high-internal-phase emulsion (HIPE) has a volume fraction of the internal phase (or dispersed phase) of at least 74.05% of the whole emulsion [1,7]. The resultant poly(HIPE) often possesses an ordered and interconnecting pore structure, and also shows certain flexibility [4]. Moreover, the high porosity of the polymer can enhance the adsorption rate of target objects, and the interconnected pores generated on the void wall can speed up the mass transfer process [1]. However, organic solvents are used as the internal phase in an O/W emulsion, especially in HIPEs, which subsequently present an environmental problem due to organic residuals in the poly(HIPEs). In particular, this problem often arises in the preparation of supermacroporous hydrophilic materials [8]. Therefore, a green emulsion template could be used to solve this issue. Zhang et al. used CO_2_ to replace the conventional oil phase as the internal phase in HIPE [9]. Organic solvents can be replaced by CO_2_ because the latter is abundant, nontoxic, nonflammable, and readily available; thus, it should be deeply utilized [10]. Cooper et al. reported poly(vinyl alcohol) hydrogel obtained from a CO_2_-in-water (C/W) emulsion template; the gel had a bulk density of 0.043 g/cm^3^, which could be useful in biomedical applications [11]. In our previous studies, UiO-66 and HKUST-1 were applicable as key emulsion stabilizers to stabilize C/W HIPEs, and the resulting composite gel had good adsorption and hydrophilicity, while the mechanical properties of the material were improved, e.g., resilience [12,13]. However, given the emulsion stability of C/W HIPEs, ionic monomers have rarely been researched in previous studies [1,7]. Polyelectrolyte gels obtained through ionic monomer polymerization have a wider range of application requirements; therefore, this challenge still needs to be overcome. Furthermore, chitosan oligosaccharides (COSs) and their derivatives are widely used in adsorptive materials and biochemical engineering due to their special structure, renewable nature, and biocompatibility [14].

To solve the above engineering problems and develop a green synthesis method for porous ionic gels, a porous sulfonated hydrogel was designed in the present work. Herein, a chitosan-oligosaccharide-based ionic monolithic hydrogel with high porosity and adjustable pore size was developed using the green C/W high-internal-phase emulsion template, thereby avoiding both the residues of organic solvents and the disadvantages of unsustainable materials. The effect of the synthesis conditions on the porous structure, equilibrium swelling ratio, adsorption capacity, and mechanical properties was investigated. Moreover, the COS-based porous hydrogel was examined for its adsorption of tetracycline (TC) to estimate its column adsorption performance.

## 2. Materials and Methods

### 2.1. Materials

Poly(vinyl alcohol) (PVA, Mw of 27,000 g/mol) and sodium 4-styrenesulfonate (SSS, ≥98% purity) were supplied by Macklin Co. (Shanghai, China). CO_2_ (>99.995% purity) was supplied by Kangdi Co. (Urumqi, Xinjiang, China). Acrylamide (AM, ≥98.0% purity) was supplied by Tianjin Yongcheng, cyclohexane was supplied by Tianjin bailing chemistry Co. (Tianjin, China), chitosan oligosaccharides (COSs, deacetylation ≥95%, viscosity 100–200 mPa·s, MW: 161.16 g/mol) were supplied by Aladdin Co. (Shanghai, China), *N*,*N*′-methylene bis(acrylamide) (MBA, ≥98.0% purity) was supplied Tianjin chemistry Co. (Tianjin, China), potassium persulfate (KPS, ≥99.5% purity) was supplied by Tianjin tienda company Co. (Tianjin, China), and tetracycline (TC) was supplied by Aladdin Co. (Shanghai, China). 

### 2.2. Preparation of the Monolith

**Figure 1:**
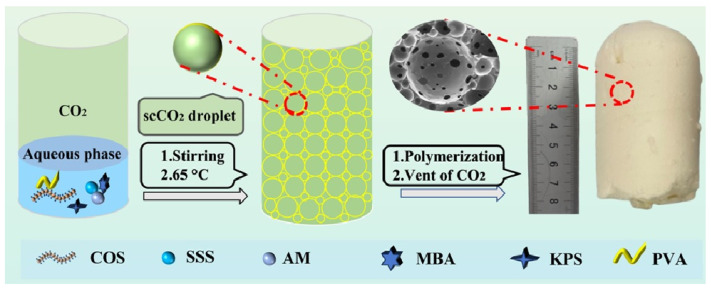
Schematic for preparation of the porous sulfonated hydrogel.

Before placing the sealed reactor in an ice-water bath, PVA, COS, AM SSS, MBA, and KPS were accurately weighed and fully dissolved in a reaction tank containing 15 mL of water. Next, the air in the reaction kettle was purged with a small amount of CO_2_ before further infusing additional CO_2_. Finally, the reaction kettle was placed in a magnetic stirrer at a constant speed of 1000 rpm for 2 h to form C/W HIPEs composed of water-soluble monomers, which were polymerized at 65 °C for 12 h while stirring. When the reactor cooled to room temperature upon completion of the reaction, the pressure-reducing valve was gently opened to remove the carbon dioxide from the kettle, followed by the reactor being opened, which yielded a monolithic COS/P(AM-co-SSS). The synthesis mechanism is depicted in Figure 1; the obtained sulfonated COS/P(AM-co-SSS) monoliths are denoted as SHCx, where Cx denotes the COS dosage. The studied samples were prepared using the controlled-variable method by varying only the polymerization pressure or the amount of COSs as described above. The raw material dosages of batch reactions are detailed in Table 1.

### 2.3. Characterization

The samples were mixed with potassium bromide and pressed into tablets for chemical structural analysis via FTIR spectrometry (Vertex 70, Bruker, Billerica, MA, USA), with a resolution of 4.0 cm^−1^. The surface morphology of the sample was examined using a Hitachi S4800 scanning electron microscope (Tokyo, Japan). The monolith was freeze-dried before being sliced into tiny slices using a blade, attached to conductive adhesive tape, then coated with gold under vacuum, and ultimately examined at a voltage of 20 kV. 

### 2.4. Swelling Behavior

The swelling ratios of the porous hydrogels were measured using a gravimetric method. The pre-weighed and dried samples were first soaked in PBS solutions of different pH at 25 °C for 5 h. Then, the samples were removed from the solution, and the excess water was gently removed from the surface with filter paper. It was finally weighed and measured. Parallel experiments were performed in triplicate and the average value was selected. The swelling ratios of the hydrogel were calculated using the following Equation (1) [7]:

(1) $$\mathrm{Swelling}\;\mathrm{ratio}=\frac{w_{e}-w_{d}}{w_{d}}$$

where *W_e_* and *W_d_* are the weights of the wet and dried samples, respectively.

### 2.5. Mechanical Property

The monolith was cut into cylinders, with a diameter of 15 mm and a thickness of 20 mm, and placed on a universal testing machine (H5KT, Tinius Olsen, Redhill, UK) for compression testing, while the compression rate measured at 5 mm/min. Finally, each sample was repeated three times to decrease error. The sample with the greatest mechanical characteristics was chosen for 50 cycles of compression testing.

### 2.6. Adsorption Process

The adsorption capacity of the monolith was studied using tetracycline as a representative of antibiotics. During the adsorption process, the initial concentration of the tetracycline solution was 200 mg/L. The adsorption properties of TC were determined using a UV-Vis spectrophotometer (UV-2250) at 355 nm. The adsorption capacity of the monolith was defined using the following Equation (2) [7]:

(2) $$Q_{e}=\frac{\left(C_{0}-C_{e}\right)\times V}{m}$$

where *V* (L) is the volume of the solution, *C*_0_ and *C_e_* (mg/L) are the concentrations before and after adsorption, respectively, and *m* (g) is the applied mass of the monolithic gel.

## 3. Results and Discussion

### 3.1. Characterization of COS-Based Monoliths

**Figure 2:**
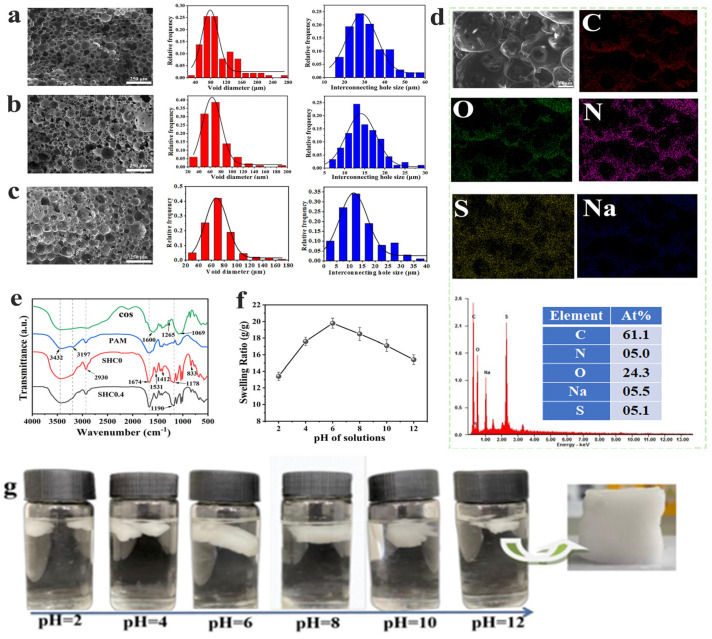
(**a**) SEM graph and pore size distribution of SHC0.1 (10 ± 2 MPa), (**b**) SEM graph and pore size distribution of SHC0.1 (12 ± 2 MPa), (**c**) SEM graph and pore size distribution of SHC0.4, (**d**) EDS mapping of SHC0.4, (**e**) FTIR spectra of COS, PAM, SHC0, and SHC0.4, (**f**) dwelling performance of samples at different pH conditions, and (**g**) photos of the hydrogel after swelling equilibrium.

The chemical composition of the material was analyzed using FTIR spectroscopy. As depicted in Figure 2e, for COSs, the broad band at 3432 cm^−1^ was due to the O–H bond stretching vibrations; and the absorption peak at 1600 cm^−1^ was due to –NH_2_ stretching; the one at 1265 cm^−1^ was the C–O–C antisymmetric stretching; and 1069 cm^−1^ was attributed to C–O stretching vibrations on the COS skeleton. The peaks at 3432 cm^−1^ and 3197 cm^−1^ in PAM were attributed to O–H and N–H stretching vibrations, respectively. The absorption peak at 1674 cm^−1^ in SHC0 was attributed to the stretching vibration of the amide carbonyl group of PAM; the absorption peak at 2930 cm^−1^ was attributed to the asymmetric stretching vibration of –CH_2_ on the benzene ring. The absorption peaks at 1531 cm^−1^ and 1412 cm^−1^ were the backbone vibration peaks of the benzene ring; the peak at 1178 cm^−1^ was the symmetric and asymmetric stretching vibration of the –SO_3_^−^ group [15,16]. For SHC0.4, the peak at 1190 cm^−1^ was the symmetric and asymmetric stretching vibration associated with the –SO_3_^−^ group, which was attributed to the formation of hydrogen bonds between the sulphonate groups in SHC0 and the –NH_2_ or –OH groups of the chitosan [17].

Furthermore, the material’s porous structure plays an important role in the practical applications of biochemical engineering and adsorption. For example, more adsorption sites were made possible through the porous structures, while the interconnected pore structure facilitated the diffusion of toxins within it, thereby increasing the adsorption capacity of the material. In these monolithic materials, the cavity formed by the removal of CO_2_ droplets was defined as the voids; the interconnected pores in the cavity walls were defined as the interconnecting pores. Figure 2a,b show the SEM graphs and the pore size distribution of SHC0.1 (obtained from two pressures), respectively; it can be clearly seen that as the CO_2_ pressure increased, the pore size of the voids and the interconnecting pores decreased significantly. The reasons may be due to the high density of the internal phase facilitating the formation of smaller emulsion droplets and homogeneous emulsion droplets. This trend was consistent with that reported by Cooper et al. [11]. The pore sizes of both voids and interconnecting pores decreased significantly with increasing CO_2_ density. Notably, when the internal phase CO_2_ was 30 g (10 MPa), the average pore size of the voids was 78.5 ± 3.0 μm and the average pore size of the interconnecting pores was 28.7 ± 0.8 μm. When the amount of internal-phase CO_2_ increased to 40 g (12 MPa), the average pore size of the voids decreased to 63.7 ± 0.9 μm and the interconnecting pores decreased to 14.1 ± 0.3 μm. Therefore, the internal phase density can be adjusted to control the pore size, which in turn can adjust the adsorption rate of the gel. 

In addition, the increase in COS content to 0.4 g resulted in an average pore size of 67.7 ± 0.4 μm for the voids and 11.8 ± 0.2 μm for the interconnecting pores, which was attributed to a reduction in emulsion droplet size following the increase in COS dosage. (Figure 2c). The inhomogeneous size of the voids may be due to the stability decreases in the emulsifier PVA, resulting in the aggregation of small droplets into larger droplets and the formation of variable-size pores in polyHIPE; similar patterns have been reported. Moreover, the pore openings of COS/P(AM-co-SSS) HIPE are more limited than those of the homopolymer obtained from acrylamide; this can be attributed to the nature of the monomer and the CO_2_ internal phase content [7]. Figure 2d shows an elemental mapping image of the SHC0.4 porous polymer surface, which clearly shows that the elements C, N, O, Na, and S were evenly distributed on the pore surface, which facilitated the adsorption of tetracyclines.

### 3.2. Swelling Property

The swelling degree of the porous SHC0.4 hydrogel at different pH solutions was investigated and the result is depicted in Figure 2f. At solution pH < 4, the anionic components are protonated, the hydrogen bonding forces between the anions and molecular chains increase, while the electrostatic repulsion between the groups is altered after the protonation of the anionic groups, resulting in an enhanced physical crosslinking, and leading to a decrease in the swelling ratio of the hydrogel [18]. At pH 4~8, the anionic sulphonic acid was completely ionized, and hydrogen bonding interactions and electrostatic repulsion were relatively weak, resulting in a swelling ratio of up to 19.9 g/g at pH = 6. At pH > 8, the solution’s ionic osmotic pressure decreased, the charge shielding effect increased, and the swelling capacity of the polymer was subsequently reduced (Figure 2g). Compared to other samples, SHC0.4 had a high swelling rate and porous structure, as well as high dimensional stability. Therefore, it shows potential applications in multiple applications, such as pharmaceutical adsorption–separation.

In addition, the solidified sulfonated gel occupied 100% of the reactor volume. After polymerization, a monolithic sample with high crosslinking was directly obtained by venting CO_2_ (Figure 1b). When the sample was immersed in water, its original shape was still retained and achieved an opaque state, as depicted in Figure 2g. Therefore, the porous hydrogel prepared through the present route was very different from those formed via the conventional synthesized method [19]. In particular, the gel was soft and dimensionally stable in this studied case.

### 3.3. Mechanical Properties of the Monoliths

**Figure 3:**
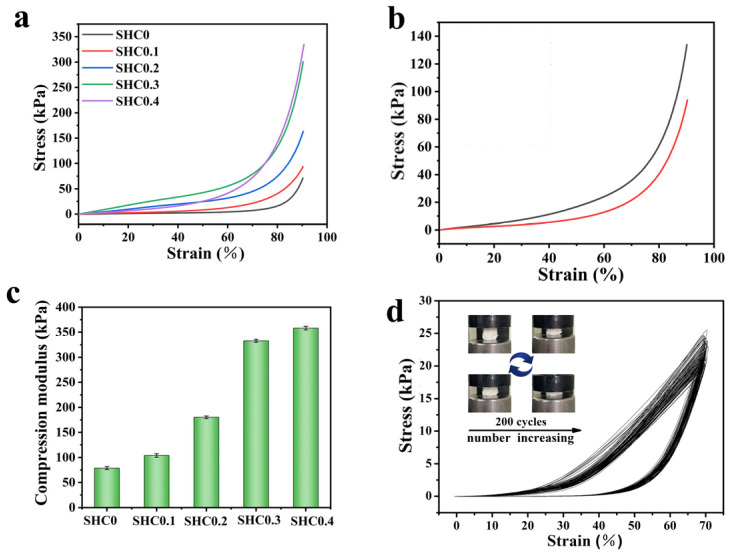
(**a**) Compressive stress–strain curves of monoliths with different COS contents, (**b**) compressive stress–strain curves of monoliths with different CO_2_ pressures (SHC0.1, the black line represents the reaction completed at 12 MPa, and the red line represents 10 MPa); (**c**) comparison of compression modulus of monoliths; (**d**) SHC0.1 stress–strain curves from 200 compression cycles.

The material must have robust mechanical properties depending on its practical significance in column separation; otherwise, displacement or fragmentation of the adsorbed material may occur during the adsorption process [20]. To explore the mechanical characteristics of the directly obtained monoliths from C/W emulsion, the modulus of elasticity was calculated using the slope at 40% strain in the stress–strain curves. As depicted in Figure 3a, the mechanical properties of the monolith were enhanced with increasing the amount of COS, which may be attributed to the fact that COS, as a natural polysaccharide, has a certain viscosity to stabilize the emulsion, while COS contains abundant –OH and –NH_2_ which can bond with PVA molecules to form hydrogen bonds, and resulted in an increase in the compressive properties of the monolith. Accordingly, the maximum stress of SHC_0.4_ could reach 334 kPa and a maximum strain of 91 %. Under the same concentrations of COS, the higher AM/SSS monomer ratio was beneficial for improving the mechanical properties of the monolith, which may be attributed to the fact that PAM could enhance the hydrogen bonding action between the polymer chains and intermolecular regularity [7,21].

Moreover, the mechanical strength of the monolith increased with increasing the CO_2_ internal phase density, and the monolith possessed a maximum stress of 131.5 kPa at a strain of 90.2% (Figure 3b). The result was ascribed to the increase in CO_2_ internal phase density, which reduces the pore size of the monolith, resulting in an ability to withstand higher pressure loads [1,7]. As depicted in Figure 3c, when COS dosages were increased from 0.3 g to 0.4 g, the compressive modulus of the monolith increased from 333.2 kPa to 367.8 kPa. This trend of material strength variation may be due to the enhanced viscosity of the emulsions, resulting in pore deformation. Nevertheless, the good elasticity and toughness of the material were reflected, and the stress–strain curves of SHC_0.1_ are depicted in Figure 3d. After 200 cycles of compression at 70% strain, it could still recover its original shape without cracking (Video S1), and the as-prepared monolith reflected favorable mechanical properties compared to those of polysaccharide-based composite aerogel [22]. At the same time, the test result exhibited a typical stress–strain curve for the foam materials. This mechanical feature facilitates its application in column separation and elastic sensors.

### 3.4. Adsorption Properties

**Figure 4:**
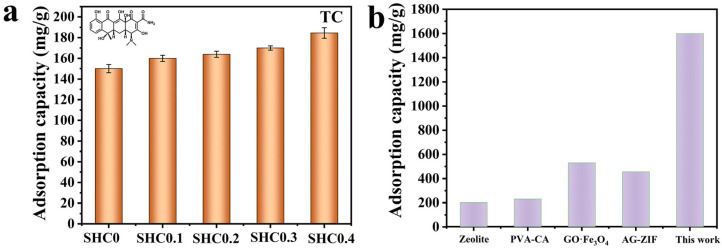
(**a**) Adsorption capacity of TC with different samples. All values are expressed as mean ± SD (n = 3). (**b**) Adsorption capacity of various adsorbents for TC.

As depicted in Figure 4, for the monolith, the adsorption of TC increased with increasing levels of PSSS and COS. Among them, SHC0.4-adsorbed TC could reach about 184.6 mg/g, which may be attributed to the synergy of the multiple interaction forces and the large pore size structure, which gave the monolith a good adsorption capacity. In particular, the N atoms of the tertiary amine groups in the TC molecule were protonated in the solution, giving a positive charge on the surface of the TC molecule. The increase in the PSSS component led to an increase in –SO_3_H groups, giving the monolith a high density of negative charges and providing more adsorption sites, while the COS contains abundant amine and hydroxyl group within the chains, also providing adsorption sites [7].

**Figure 5:**
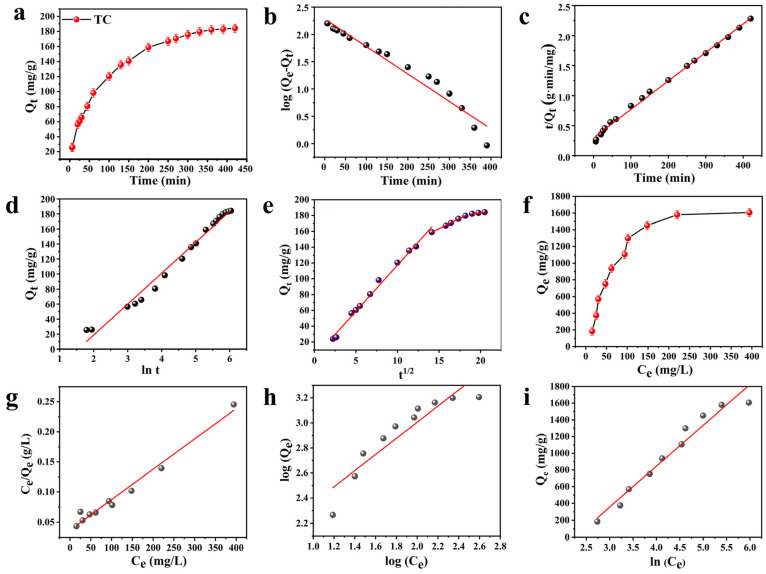
(**a**) Kinetic curves of adsorption of SHC0.4 on TC at 298 K temperatures, (**b**) linear plots of the proposed first-order kinetic model, (**c**) linear plots of the pseudo-second-order kinetic model, (**d**) line plots of the Elovich model, (**e**) line plots of the particle diffusion model, (**f**) isotherm curves of adsorption of SHC0.4 on TC at 298 K temperature, (**g**) line plots of the Langmuir isotherm, (**h**) line plots of the Freundlich isotherm, and (**i**) line plots of the Temkin mode.

To systematically investigate the kinetics and isothermal adsorption of TC by the SHC0.4 monolith, pseudo-first-order, pseudo-second-order, Elovich, and intraparticle diffusion kinetic models as well as Langmuir, Freundlich, and Temkin isothermal adsorption models were subsequently selected to fit the data. As can be seen from Figure 5 and Table 2, the adsorption and intraparticle diffusion adsorption processes were consistent. The pseudo-second-order kinetic model provided a better description of the toxin adsorption process, indicating the presence of internal diffusion in the adsorption process and the presence of chemisorption in the adsorption process. Moreover, the mechanism of toxin adsorption was best explained by the Langmuir isothermal adsorption model during the isothermal adsorption process of the monoliths, when the toxin interacted with the monolith surface [23]. The toxin interacted with the active adsorption sites on the surface of the monolith and formed a homogeneous single-molecule adsorption layer.

**Figure 6:**
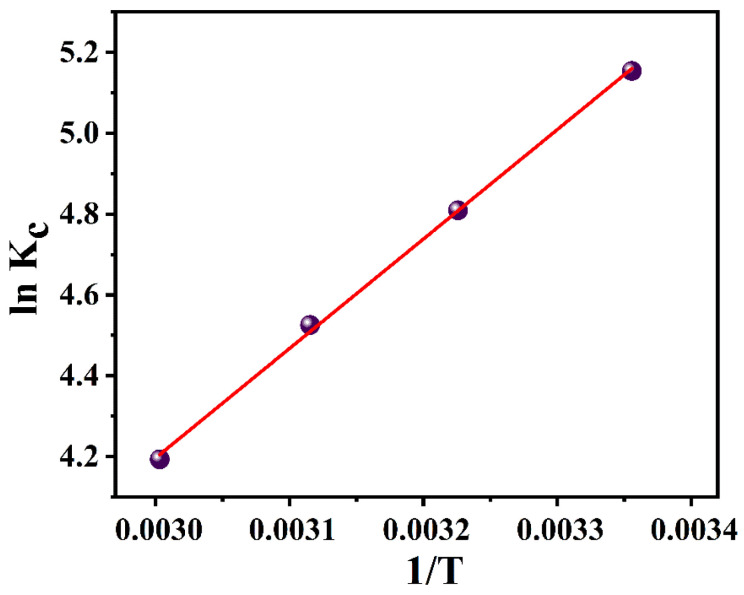
Linearity of lnKc versus 1/T for adsorbent SHC0.4.

Furthermore, the adsorption of TC by the monolith was found to decrease with increasing temperature, indicating that the process is exothermic. By expressing the Van’t Hoff equation as a linear plot of lnKc versus 1/T, ΔH and ΔS were calculated from the slope and intercept [24]. As depicted in Figure 6 and Table 3, ΔG < 0 indicates that the adsorption of TC by SHC0.4 is a spontaneous process as it is thermodynamically favorable. ΔH was estimated to be −22.40 kJ/mol (ΔH < 0), indicating that the adsorption process is exothermic due to physical adsorption. Therefore, the adsorption decreased with increasing temperature |ΔH| > |TΔS|, indicating that the adsorption of TC by SHC0.4 is an enthalpy-driven process. To some extent, the increase in the enthalpy change may reflect the type of interaction in the adsorption process. Physical adsorption, such as van der Waals interactions and hydrogen bonding, is usually below 20 kJ/mol [24]. Electrostatic interactions range from 20 to 80 kJ/mol and are often referred to as physical adsorption. Chemisorption bonds are usually in the range of 80 to 450 kJ/mol. Therefore, a ΔH of −22.40 kJ/mol indicates strong interactions between TC and SHC0.4, with electrostatic interactions being the main force in the TC adsorption process.

### 3.5. State-of-the-Art Comparison of the TC Adsorbents

Recently, different kinds of adsorbents for toxin removal have been reported. To further indicate the applicability of the research, an in-depth comparison of some studied materials is collected and depicted in Figure 4b. The monolith prepared in this study showed good adsorption. It has a limited ability to absorb TC using Zeolite [25] and GO [26]. As depicted in Figure 4b, in terms of TC removal, the monolith (SHC0.4) showed optimal adsorption capacity for TC [18,27,28]. As a result, the monoliths described above have potential applications in such antibiotics separation applications.

## 4. Conclusions

In summary, a polysaccharide-derived monolithic hydrogel with highly interpenetrating porous structures was obtained using a green C/W HIPEs. In the presence of a COS, the COS/P(AM-co-SSS) monolith had typical pore throats of 28 μm and exhibited strong mechanical stability. Furthermore, the monoliths exhibited high toxin and antibiotic removal ability due to the synergistic effect of the specific pore structure and the surface active sites within the monolith. The chitosan molecular chain in the obtained gel is rich in amines and hydroxyl groups, which can provide active sites for the adsorption of TC. When the molar ratio of the two monomers was fixed and the COS content was higher than 0.3 g, the adsorption capacity of the gel had the optimum value. Thus, elastic COS/P(AM-co-SSS) hydrogel has excellent antibiotic removal ability with a 1600 mg/g for TC and its behavior suggests potential for applications in medical wastewater treatment. This synthesis strategy can be used for the polymerization of other ionic monomers in C/W emulsions and expand their application in biomedical applications. Future work will focus on the demonstration of the removal ability of a larger spectrum of pollutants using porous sulfonated hydrogels.

## Figures and Tables

**Table 1:** Detailed conditions of COS/P(AM-co-SSS) monolith *^a^*.

Entry	PVA *^b^*	COS	MBA	CO_2_	H_2_O
	(%, *w*/*v*)	(g)	(%, *w*/*v*)	(MPa)	(mL)
SHC0	5	0	8	10 ± 2	15
SHC0.1	5	0.1	8	10 ± 2	15
SHC0.1 *^c^*	5	0.1	8	12 ± 2 *^c^*	15
SHC0.2	5	0.2	8	10 ± 2	15
SHC0.3	5	0.3	8	10 ± 2	15
SHC0.4	5	0.4	8	10 ± 2	15

*^a^* Reaction conditions: monomer molar ratios =1:1; reactor volume = 100 mL, H_2_O/CO_2_ = 15:85 *v*/*v*; 65 °C, polymerization time: 12 h. Mass_(AM+SSS)_ = 4.15 g. *^b^* Weight relative to water volume. *^c^* Change reaction pressure under same conditions.

**Table 2:** Pseudo-first order, pseudo-second order kinetic model parameters, Elovich model parameters, ion diffusion model, and Langmuir, Freundlich, and Temkin isothermal adsorption model parameters for TC adsorption by SHC0.4 at 298 K temperature.

Pseudo-First-Order			Pseudo-Second-Order			Elovich		
Q_e_	K_1_	R^2^	Q_e_	K_2_	R^2^	α	β	R^2^
(mg·g^−1^)	(min^−1^)		(mg·g^−1^)	(mg^−1^·g·min^−1^)		(mg·g^−1^·min^−1^)	(g·mg^−1^)	
174.3	0.010	0.956	211.4	0.7 × 10^−3^	0.996	6.20	0.021	0.984
			Intra-particle diffusion					
K_d1_			R^2^		K_d1_		R^2^	
(mg·g^−1^·min^−1/2^)					(mg·g^−1^·min^−1/2^)			
11.68			0.992		4.95		0.992	
Langmuir			Freundlich			Temkin		
*K_L_*	*q*_m_	*R* ^2^	*K_f_*	*n*	*R* ^2^	*K_t_*	*f*	*R* ^2^
(L·mg^−1^)	(mg g^−1^)					(J·mol^−1^)		
1.37 × 10^−3^	1981.4	0.978	35.64	1.56	0.837	489.17	0.102	0.954

**Table 3:** Thermodynamic data of adsorption of TC by SHC0.4 porous gel.

T(K)	Kc	−ΔG (KJ·mol^−1^)	−ΔH (KJ·mol^−1^)	−ΔS (J·mol^−1^·k^−1^)
298	172.4	12.7	22.4	32.5
310	122.7	12.3		
321	91.8	12.1		
333	66.0	11.6		

## Data Availability

Not applicable.

## Supplementary Materials

The following supporting information can be downloaded at: https://www.mdpi.com/article/10.3390/gels9090703/s1.
